# Increasing precipitation during first half of growing season enhances ecosystem water use efficiency in a semiarid grassland

**DOI:** 10.3389/fpls.2023.1119101

**Published:** 2023-02-02

**Authors:** Jiayang Zhang, Zhongling Yang, Daiyu Qiao, Lei Su

**Affiliations:** ^1^ International Joint Research Laboratory for Global Change Ecology, Laboratory of Biodiversity Conservation and Ecological Restoration, School of Life Sciences, Henan University, Kaifeng, Henan, China; ^2^ School of Life Sciences & Basic Medicine, Xinxiang University, Xinxiang, China

**Keywords:** evapotranspiration, gross ecosystem productivity, precipitation amount, precipitation seasonality, water use efficiency

## Abstract

Precipitation amount and seasonality can profoundly impact ecosystem carbon (C) and water fluxes. Water use efficiency (WUE), which measures the amount of C assimilation relative to the amount of water loss, is an important metric linking ecosystem C and water cycles. However, how increasing precipitation at different points in the growing season affects ecosystem WUE remains unclear. A manipulative experiment simulating increasing first half (FP+) and/or second half (SP+) of growing-season precipitation was conducted for 4 years (2015-2018) in a temperate steppe in the Mongolian Plateau. Gross ecosystem productivity (GEP) and evapotranspiration (ET) were measured to figure out ecosystem WUE (WUE = GEP/ET). Across the four years, FP+ showed no considerable impact on ecosystem WUE or its two components, GEP and ET, whereas SP+ stimulated GEP but showed little impact on ET, causing a positive response of WUE to FP+. The increased WUE was mainly due to higher soil water content that maintained high aboveground plant growth and community cover while ET was stable during the second half of growing season. These results illustrate that second half of growing-season precipitation is more important in regulating ecosystem productivity in semiarid grasslands and highlight how precipitation seasonality affects ecosystem productivity in the temperate steppe ecosystem.

## Introduction

Plants assimilate CO_2_ from the atmosphere at the expense of water loss during photosynthesis (Lawson and Vialet-Chabrand, 2019). The tradeoff between plant productivity and water use can be quantified by water use efficiency (WUE), the magnitude of carbon (C) gained per unit of water consumption (Beer et al., 2009; Lawson and Vialet-Chabrand, 2019; Bai et al., 2020; Dong et al., 2021). WUE, therefore, represents the coupling of terrestrial ecosystem C and hydrologic cycles (Song et al., 2016; Kang et al., 2020; Zhang et al., 2020b) and is regarded as a vital indicator for characterizing terrestrial ecosystems in response to on-going climate change (Knauer et al., 2017; Li et al., 2018; Zheng et al., 2019). Higher WUE implies that plants can synthesize more C by consuming less water resources (Tarin et al., 2020). Therefore, evaluating of the dynamics of WUE can enhance our understanding of regional energy and mass budgets (Li et al., 2016; Hatfield and Dold, 2019).

Ecosystem WUE is generally estimated as the ratio of gross ecosystem productivity (GEP), net ecosystem CO_2_ exchange (NEE), or gross primary productivity (GPP) to evapotranspiration (ET) (Hu et al., 2008; Guerrieri et al., 2016; Medlyn et al., 2017). GEP/ET is the most commonly used metric of ecosystem WUE (Beer et al., 2009; Niu et al., 2011; Bai et al., 2020; Volik et al., 2021). Ecosystem WUE is driven by the trade-off between GEP and ET, and thus biotic and climatic factors that affect C assimilation or water loss or both could cause changes in WUE (Leonardi et al., 2012). GEP can be regulated by climate change-induced shifts of limiting resources, as well as variation in species composition because plant species differ in their WUE (Roman et al., 2015). ET is routinely partitioned into vegetation transpiration and soil evaporation, these two components are likely to respond differently to changing environment (Hu et al., 2009; Niu et al., 2011; Yimam et al., 2015; Li et al., 2016; Medlyn et al., 2017).

Climate change is dramatically altering precipitation magnitude and timing in ecosystems globally (Trenberth, 2011; Bernacchi and VanLoocke, 2015; Konapala et al., 2020). Natural ecosystems, especially arid and semiarid regions that are often limited by water availability, are highly sensitive to both precipitation amount and timing (Jongen et al., 2011; Yang et al., 2020). Furthermore, the water requirements of plants can differ greatly throughout the growing season and can be species-specific (Denton et al., 2017). Changes in precipitation magnitude and seasonality are anticipated to alter ecosystem C assimilation and water loss in various ecosystems. For example, both precipitation magnitude and timing can profoundly influence ecosystem productivity (Robinson et al., 2013) and alter ecosystem C and water cycles, with consequent impacts on ecosystem WUE (Eamus et al., 2013; Yang et al., 2016; Zheng et al., 2019).

In arid and semiarid grasslands, water is a key limiting factor restricting ecosystem productivity and ecosystem functioning (Huxman et al., 2004; Gao et al., 2016; Zhang et al., 2021), making C-water relationships are highly subject to shifts in precipitation regime. Substantial variations in both precipitation magnitude and seasonality have been documented in the temperate steppe (Fang et al., 2005; Xu and Wang, 2016). WUE is a crucial indicator of ecosystem productivity, and monitoring and evaluating variation in WUE may provide valuable information for exploring the responses of ecosystem functions to changes in precipitation regimes. Therefore, there is a compelling need to understand how ecosystem WUE responds to changes not only in precipitation amount but also in precipitation timing. As part of a field experiment simulating changing precipitation started in April 2015, this study was designed to investigate the responses of ecosystem WUE to increasing first half and/or second half of growing-season precipitation. The aims of this study were 1) to examine how ecosystem WUE responds to increasing growing-season precipitation, 2) to access which period of precipitation increase is more decisive in determining ecosystem WUE and 3) to identify the factors controlling ecosystem WUE under higher growing season precipitation.

## Materials and methods

### Study site

This study was performed at the Ecological Restoration Experimental Site of Duolun County (1324 m a.s.l., 42°02′N, 116°17′E), a typical temperate steppe of the southern margin of the Mongolia Plateau. The mean annual air temperature and precipitation were 2.4°C and 382.2 mm, respectively. At this site, 90% of the annual precipitation falls between April and September. The potential evaporation, estimated by the Penman-Monteith equation using the original data acquired from Duolun meteorological station, ranged from 620 mm to 1416 mm (Li and Zhou, 2016). The potential evaporation greatly exceeds precipitation, indicating the study site is water-limited area. The hottest and coldest months are July (mean monthly temperature is 19.1°C) and January (mean monthly temperature is -17.3°C), respectively. Six perennial species including *Stipa krylovii*, *Agropyron cristatum*, *Potentilla acaulis*, *Cleistogenes squarrosa*, *Allium bidentatum*, and *Artemisia frigida* comprise of more than 70% of aboveground biomass. The soil is classified as a Haplic Calcisol according to the FAO classification, with sand, silt, and clay comprising 62.75%, 20.30%, and 16.95%, respectively. The soil aggregate and capillary porosities are 57.16% and 31.10%, respectively, at the depth of 0-10 cm (Su et al., 2021). The average rooting depth is 11.6 cm.

### Experimental design

The experiment, which was established in April 2015, used a completely randomized block design. The experiment included 35 plots (4 m × 4 m) with five replicates in each of seven treatments. These treatments comprised of control (C), a 60% decrease (FP-)/increase (FP+) in precipitation during the first half of growing season (from April to June), a 60% decrease (SP-)/60% increase (SP+) in precipitation during the second half of growing season (from July to September), and a 60% decrease (P-)/increase (P+) in precipitation during the entire growing season (from April to September). The treatments of precipitation exclusion (FP-, SP-, and P-) were not included in this study. The level of 60% of ambient precipitation, both for addition and removal, was based on the historical meteorological data over the past 54 years (1961-2014). A buffer zone (width = 1.5 m) was set between neighboring plots.

**Figure 1:**
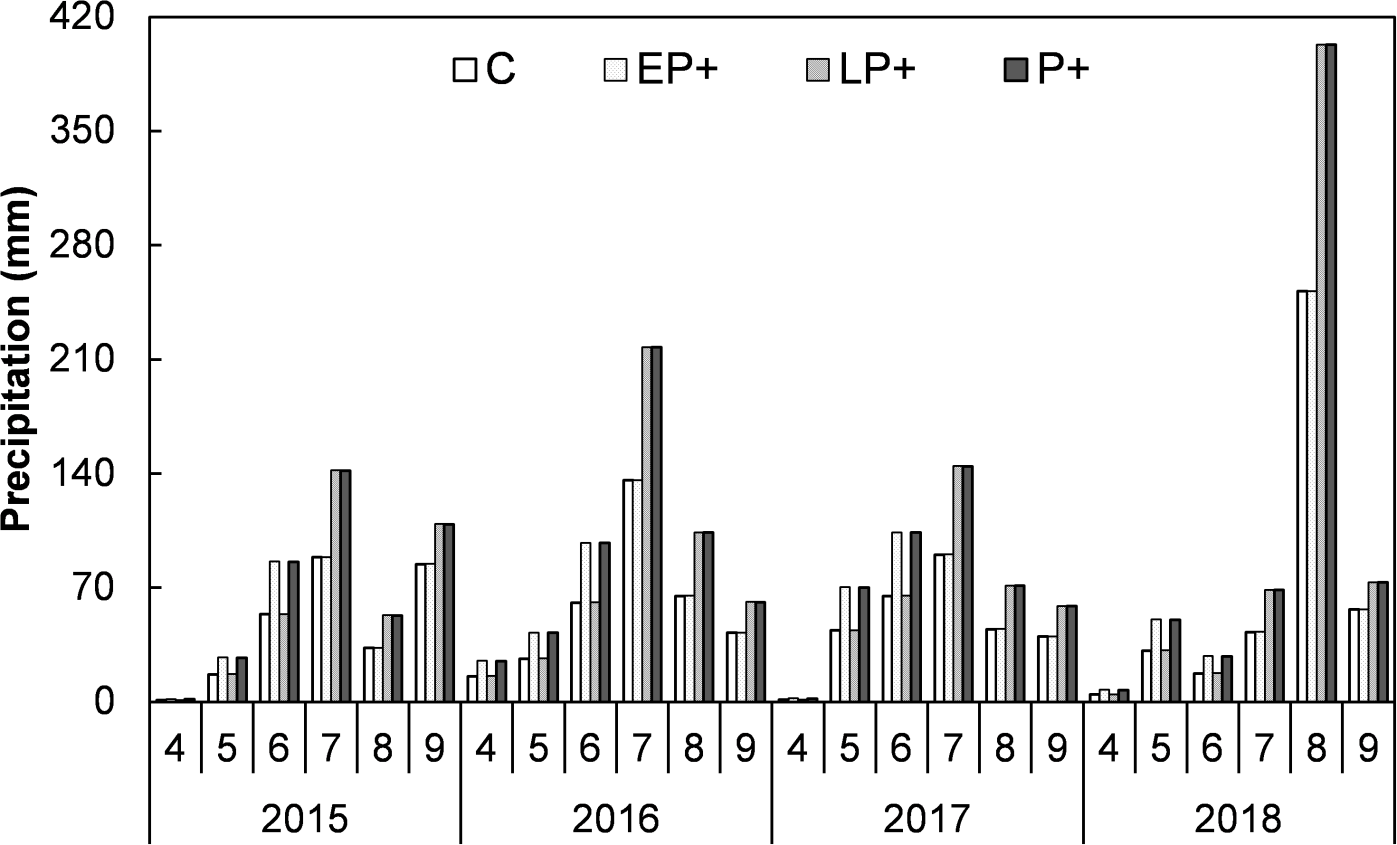
Monthly precipitation of the four treatments during the growing seasons of from 2015 to 2018. C, control; FP+, increasing first half of growing-season precipitation; SP+, increasing second half of growing-season precipitation; P+, increasing entire growing-season precipitation.

From April 15th to June 30th, the control and SP+ plots received natural precipitation while the FP+ and P+ plots received 60% additional rainwater that was applied manually and evenly with a water pipe. From July 1st to September 15th, SP+ and P+ plots received 60% additional rainwater while control and FP+ plots received ambient precipitation (Yang et al., 2020). Decreasing precipitation was controlled by slat paneled shelters. Shelters were made by organic plastic sheet. All shelters were removed after the cessation of precipitation management. These shelters followed the design of Gherardi and Sala (2013) and had little effect on temperature, wind speed, and light intensity. The highest and lowest ends of the shelters were 1.2 m and 0.5 m, respectively, the tilt angle was 30°. The size of the shelters was 4 m × 4 m. The outermost 0.5-m band inside each shelter was not sampled to avoid edge effects, while the 3.5 m × 3.5 m area at the center was used for study monitoring. The rainwater added in FP+, SP+, and P+ plots was collected from the EP-, LP-, and P- plots (**Figure 1**). The insufficient part was supplemented from collected rainwater if there was some water lost during rainwater transport process. We separated the 3.5 m × 3.5 m plot into two portions: the section (2 m × 1 m) at the center was employed for vegetation monitoring, and the other section was employed for water and C flux measurement.

The flowering phenology of common species is the basis of the division of first half (April-June) and second half (July-September) of growing season (Yang et al., 2020; Zhang et al., 2020a). The years with the worst drought in the first half and second half of growing season occurred in 2007 and 2009, respectively. The precipitation data were 59.6% and 55.8% lower than the average precipitation. The years with the maximum precipitation in the first half and second half of growing season happened in 1979 and 1983, respectively. The study site received 74.0% and 43.4% more rainfall than the historical average.

### Soil microclimate and vegetation indexes

Volumetric soil water content at a depth of 20 cm was measured using Diviner 2000 (Sentek Pty Ltd., Balmain, Australia) six times per month during the four growing seasons from 2015 to 2018.

Plant community cover was monitored in a permanent quadrat (1 m × 1 m) of each plot. To avoid edge effects, these quadrats were placed more than 0.5 m away from boundary. Measurement was carried out in early September every year when plant biomass was at its peak (Zhang et al., 2020a). The other permanent 1 m × 1 m quadrat in the same plot was clipped to measure aboveground net primary production (ANPP). The collected materials were dried at 65°C for 48 hours and weighed to determine ANPP. Root in-growth method was employed to measure belowground net primary production (BNPP). We excavated two cylindrical holes (50 cm in depth) using a 7-cm soil auger at two diagonal corners in each plot in mid-April. After removing roots and gravel (the diameter of the mesh was 2 mm), we refilled the holes with the sieved soil. The root in-growth samples were collected in October using a soil auger (5 cm in diameter) at the center of the same holes. The total weight of the oven-dried root samples was taken as the BNPP (Kong et al., 2017; Yang et al., 2020).

### Ecosystem WUE

In the present study, ecosystem WUE was determined by the division of GEP of ET. In April 2015, a permanent aluminum frame (0.5 m × 0.5 m) was inserted into the soil in each subplot to a depth of about 3 cm. A transparent chamber (0.5 m × 0.5 m in area, 0.5 m in height) affiliated to an infrared gas analyzer (IRGA; LI-6400, LiCor, Lincoln, NE, USA) was placed above the frame to measure ecosystem water and CO_2_ fluxes. Measurements were taken twice or thrice per month. Two small continuously operating electric fans were employed to mix the air inside the chamber. Nine consecutive recordings of water and CO_2_ fluxes were taken at 10-s interval during a 90-s period. ET and NEE were computed based on the time courses of water and CO_2_ fluxes. After these measurements, the chamber was covered with a black lightproof shelter to stop photosysthesis, and the CO_2_ flux was measured again to determine ecosystem respiration (ER). GEP was the difference between ER and NEE. Positive and negative NEE values refer net carbon uptake by and release from the ecosystem, respectively.

### Statistical analysis

The mean values of growing-season ET, GEP, WUE, SM, ANPP, BNPP, and plant community cover were derived from the monthly mean values from April to September. First half (FSM) and second half (LSM) of growing-season soil water contents were the mean values from April to June and from July to September, respectively.

The main and interactive effects of FP+ and SP+ on ecosystem WUE and its components were analyzed using repeated measures ANOVAs. One-way ANOVAs were employed to test the impacts of different precipitation treatments on measured parameters. The relationship between SM, vegetation indexes and ecosystem WUE, GEP and ET were analyzed with linear regression. All statistical analyses were performed with SPSS 19.0 (SPSS Inc., Chicago, IL, USA).

## Results

### Precipitation and soil moisture

**Figure 2:**
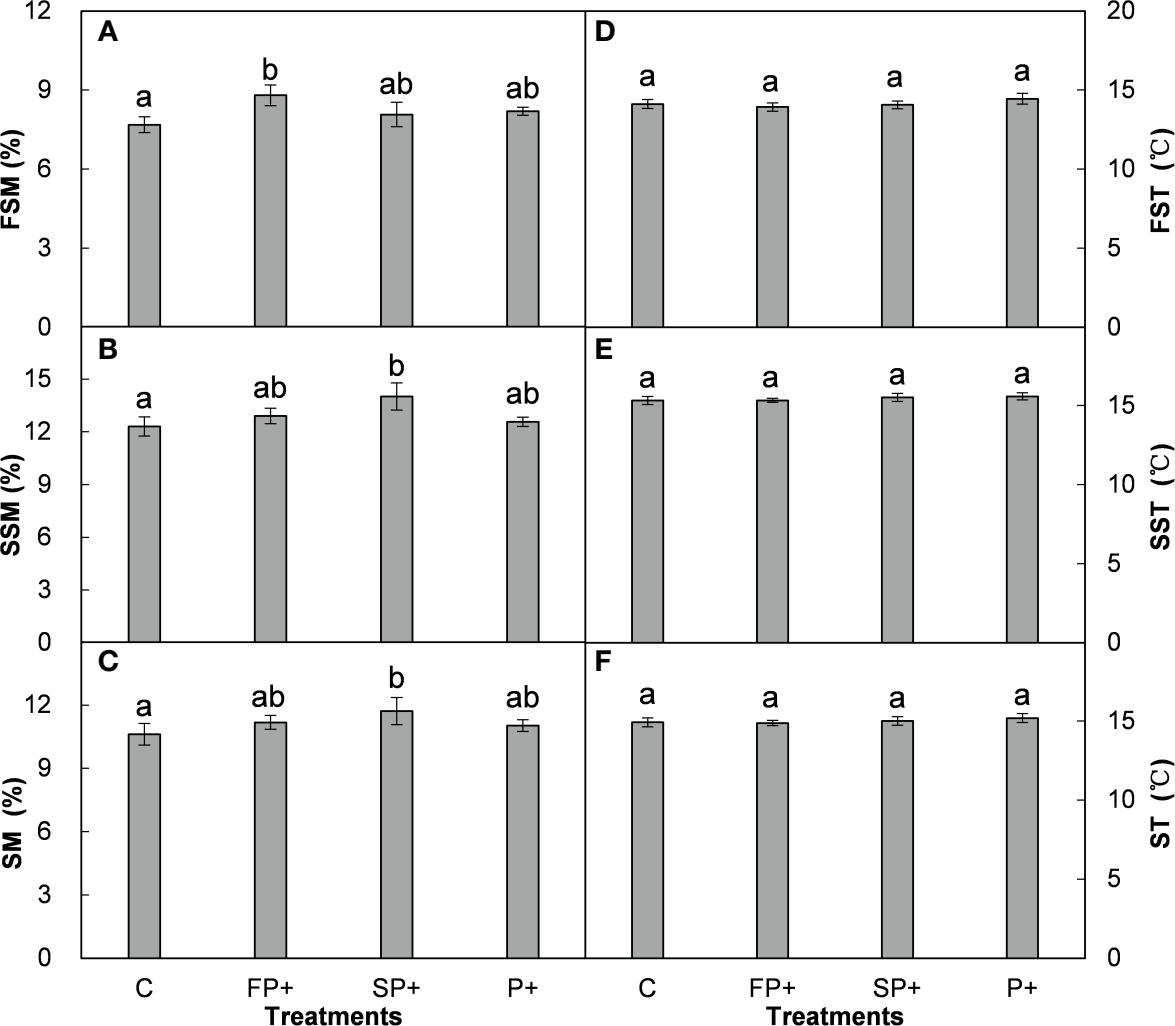
Effect of increasing first half and late growing-season precipitation on first half of (FSM) **(A)**, second half of (SSM) **(B)**, and entire (SM) **(C)** growing-season soil moisture, first half of (FST) **(D)**, second half of (SST) **(E)**, and entire (ST) **(F)** growing-season soil temperature. Different letters indicate significant differences among different precipitation treatments (*P* < 0.05), the same in **Figures 3**, **4**.

Averaged over the four years, FP+, SP+, and P+ elevated growing season precipitation amount by 50.6 mm (68.7%), 144.2 mm (59.6%), and 194.8 mm (60.0%), respectively, in comparison of the mean precipitation of the first half of, the second half of, and entire growing season of the past 54 years (1961-2014). The SP+ treatment significantly enhanced SSM by 1.71% and SM by 1.29% (absolute change, both *P* < 0.01), respectively, and marginally elevated FSM by 0.87% (*P* = 0.088; **Figures 2A–C**). However, FP+ had little effect on FSM, SSM, and SM (all *P* > 0.05). Moreover, none of FP+, SP+, and P+ showed significant influence on FST, SST, and ST over the study period (all *P* > 0.05; **Figure 2D–F**) No interactive effect of FP+ and SP+ on FSM, SSM, or SM was found (all *P* > 0.05, **Table 1**; **Figure 2**).

**Table 1:** Results (*P*-values) of repeated measures of ANOVAs on the impacts of increasing first half (April-June) and second half (July-September) of growing-season precipitation and their interactions on first half of (FSM), second half of (SSM), entire (SM) growing-season soil moisture, ANPP, BNPP, plant community cover, GEP, ET, and WUE over the four years.

	FSM	SSM	SM	ANPP	BNPP	Community cover	GEP	ET	WUE
FP+	0.714	0.974	0.748	0.400	0.143	**0.016**	0.469	0.765	0.534
SP+	0.091	**<0.01**	**<0.01**	0.225	0.358	**0.008**	**0.072**	0.902	**0.028**
FP+×SP+	0.985	0.427	0.658	0.908	0.883	0.900	0.258	0.587	0.534

The bold values highlight the significance at *P* < 0.05.

### ANPP, BNPP, and plant community cover

**Figure 3:**
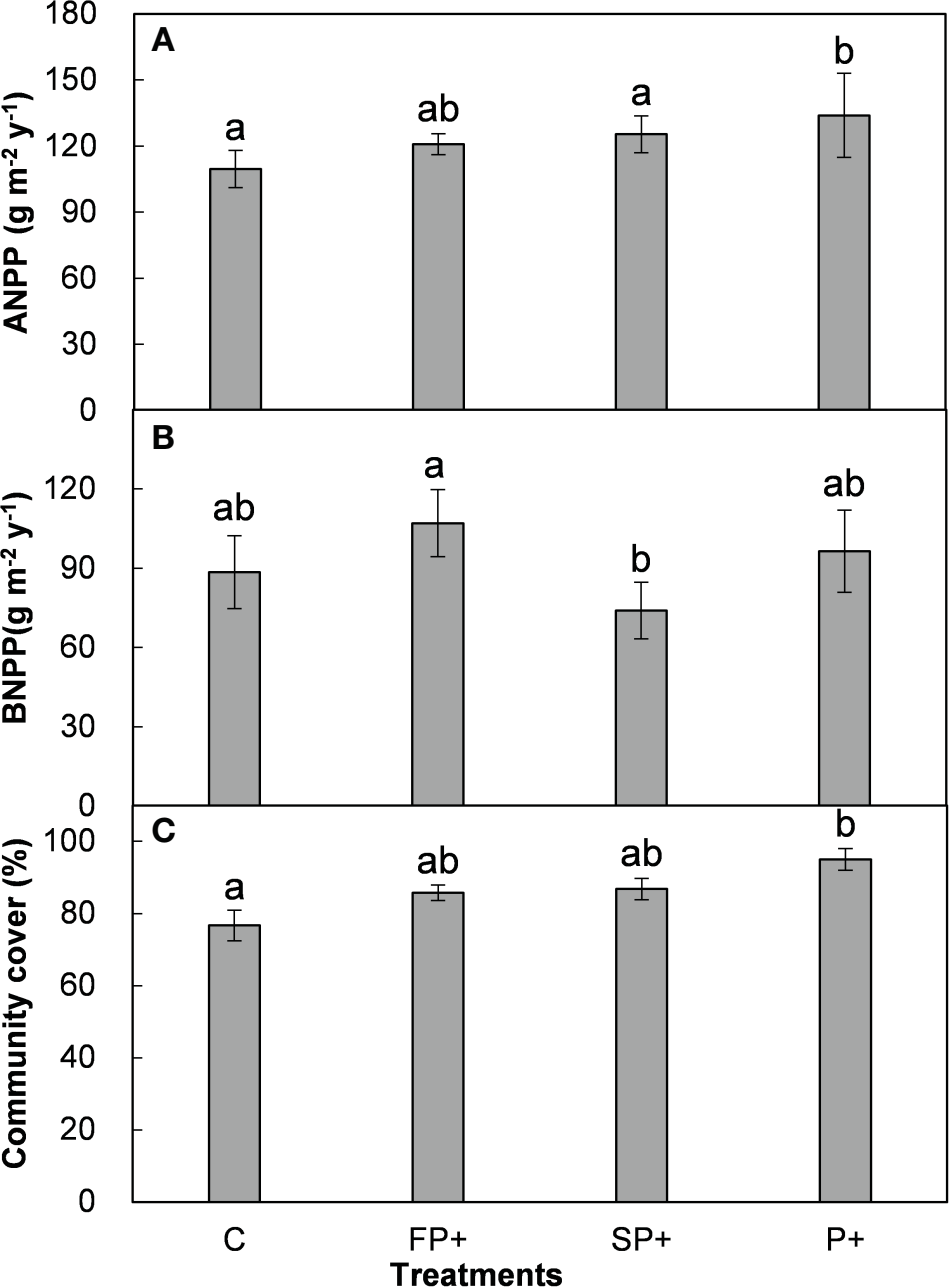
Aboveground net primary production (ANPP, **A**) and belowground net primary production (BNPP, **B**), and plant community cover **(C)** in response to increasing first half and/or second half of growing-season precipitation.

Pooling data from 2015 to 2018, FP+ stimulated ANPP and BNPP by 8.4% and 25.2%, respectively. SP+ enhanced ANPP by 12.6% but suppressed BNPP by 12.9%. Although there were large changes in ANPP and BNPP, none of these changes were significant (all *P* > 0.05, **Table 1**; **Figure 3**). FP+ and SP+ substantially enhanced plant community cover by 8.7% and 9.7% (absolute change), respectively. There was no interactive effect of FP+ with SP+ on ANPP, BNPP, or plant community cover (**Table 1**; **Figure 3**).

### GEP, ET, and ecosystem WUE

**Figure 4:**
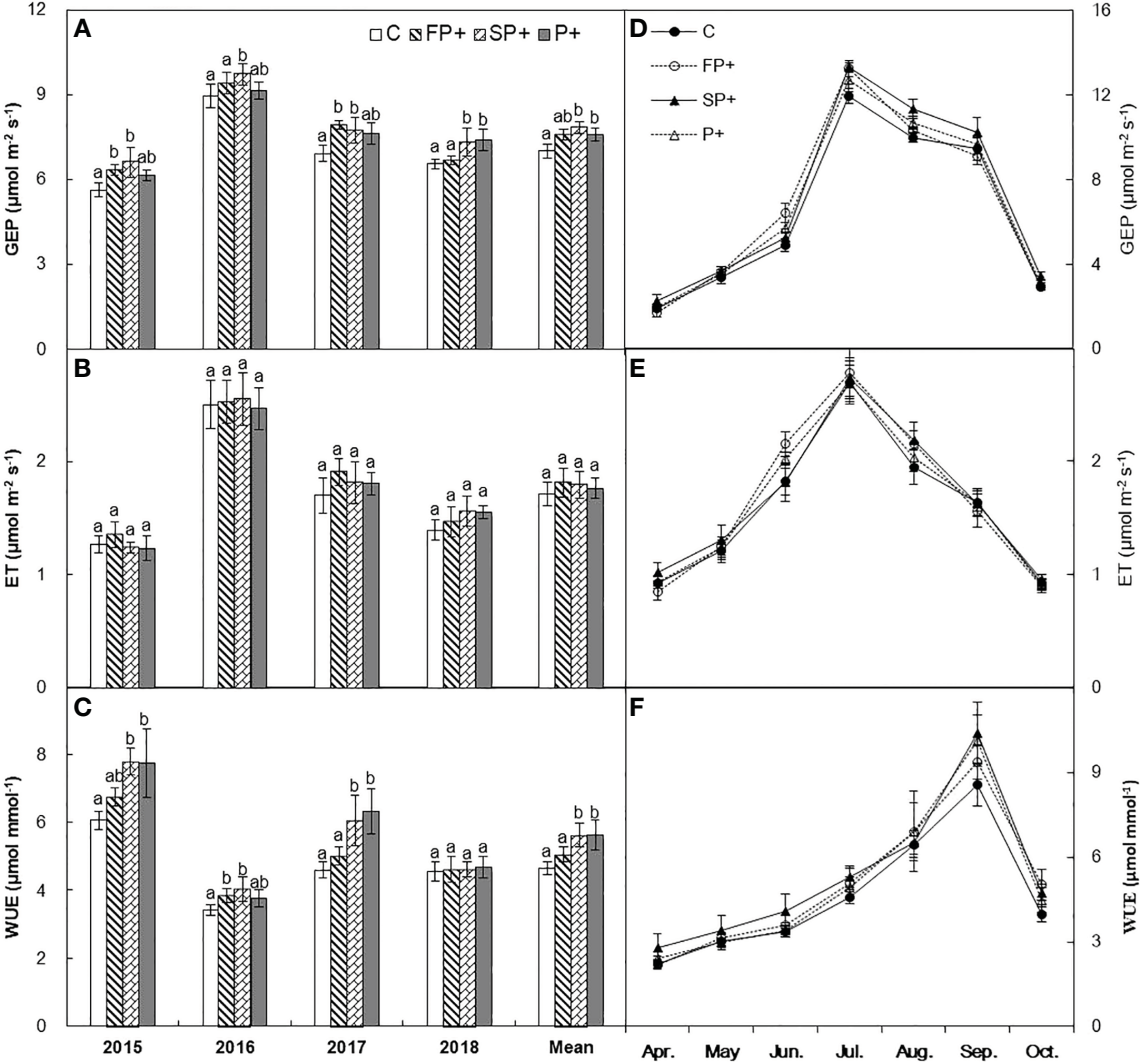
Mean and seasonal dynamics of gross ecosystem productivity (GEP) **(A, D)**, evapotranspiration (ET) **(B, E)**, and ecosystem water use efficiency (WUE) **(C, F)** in response to increasing first half and/or second half of growing-season precipitation.

Intense intra-annual variability in GEP, ET, and WUE was detected in **Figure 4**. GEP and ET were lowest in April, and reached their maxima in July, and then declined in August and September. WUE was also lowest in April, and then increased in the spring and summer, and peaked in September (**Figures 4D, E**). Averaged over the four years, growing season GEP, ET, and WUE did not respond to FP+ (all *P* > 0.05, **Table 1**). SP+ marginally enhanced GEP by 4.8% (*P* = 0.072), but it did not affect ET (*P* > 0.05). WUE was significantly stimulated by 14.8% under the SP+ treatments (*P* < 0.05, **Table 1**; **Figures 4A–C**). No interactive effect of FP+ and SP+ on GEP, ET, or WUE was detected (**Table 1**; **Figure 4**, all *P* > 0.05).

### Relationship of ecosystem WUE with its driving factors

**Figure 5:**
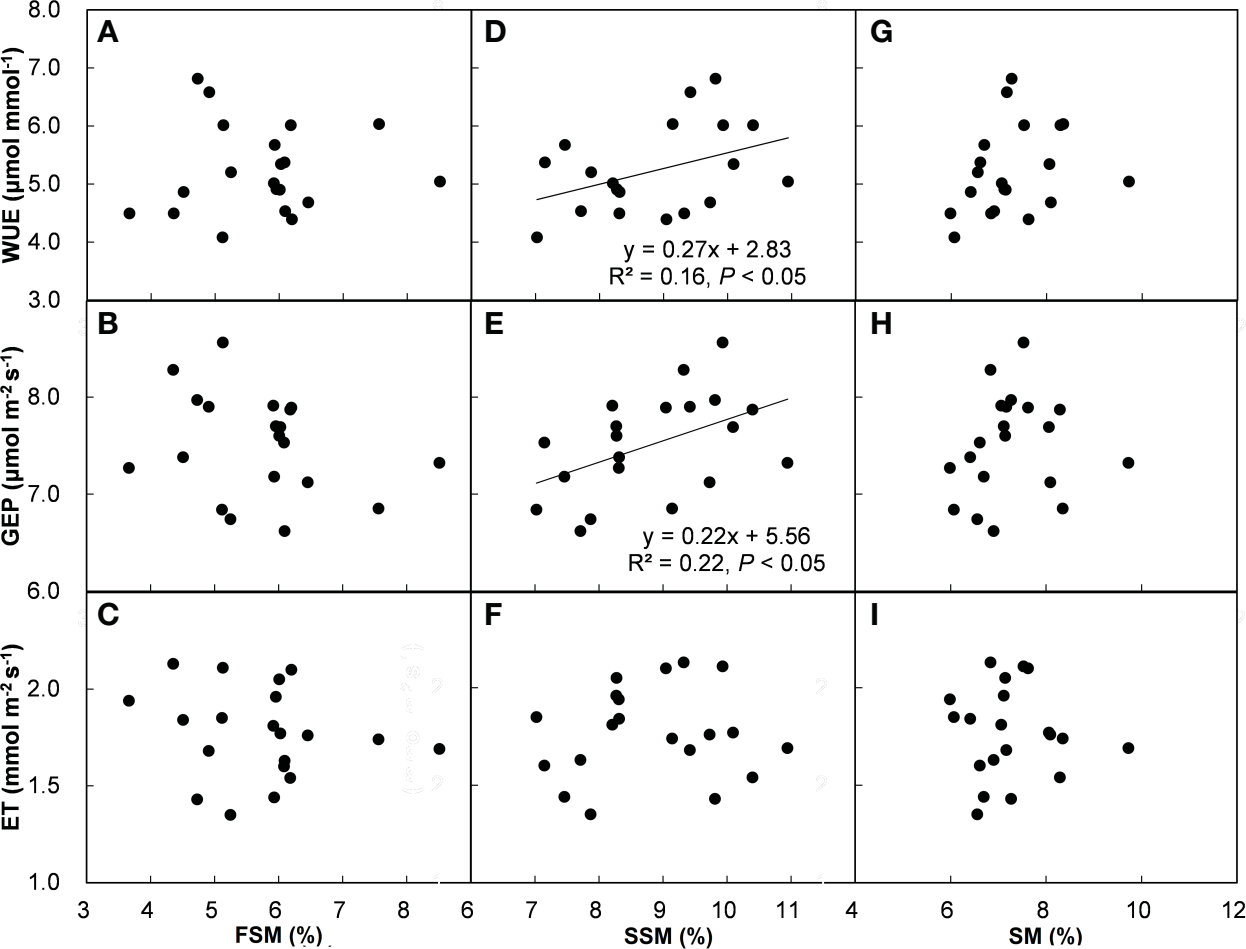
Relationships of WUE, GEP, and ET with first half of (FSM) **(A–C)**, second half of (SSM) **(D–F)**, and entire (SM) **(G–I)** growing-season soil water content. Each point represents the average of each plot, the same in **Figure 6**.

**Figure 6:**
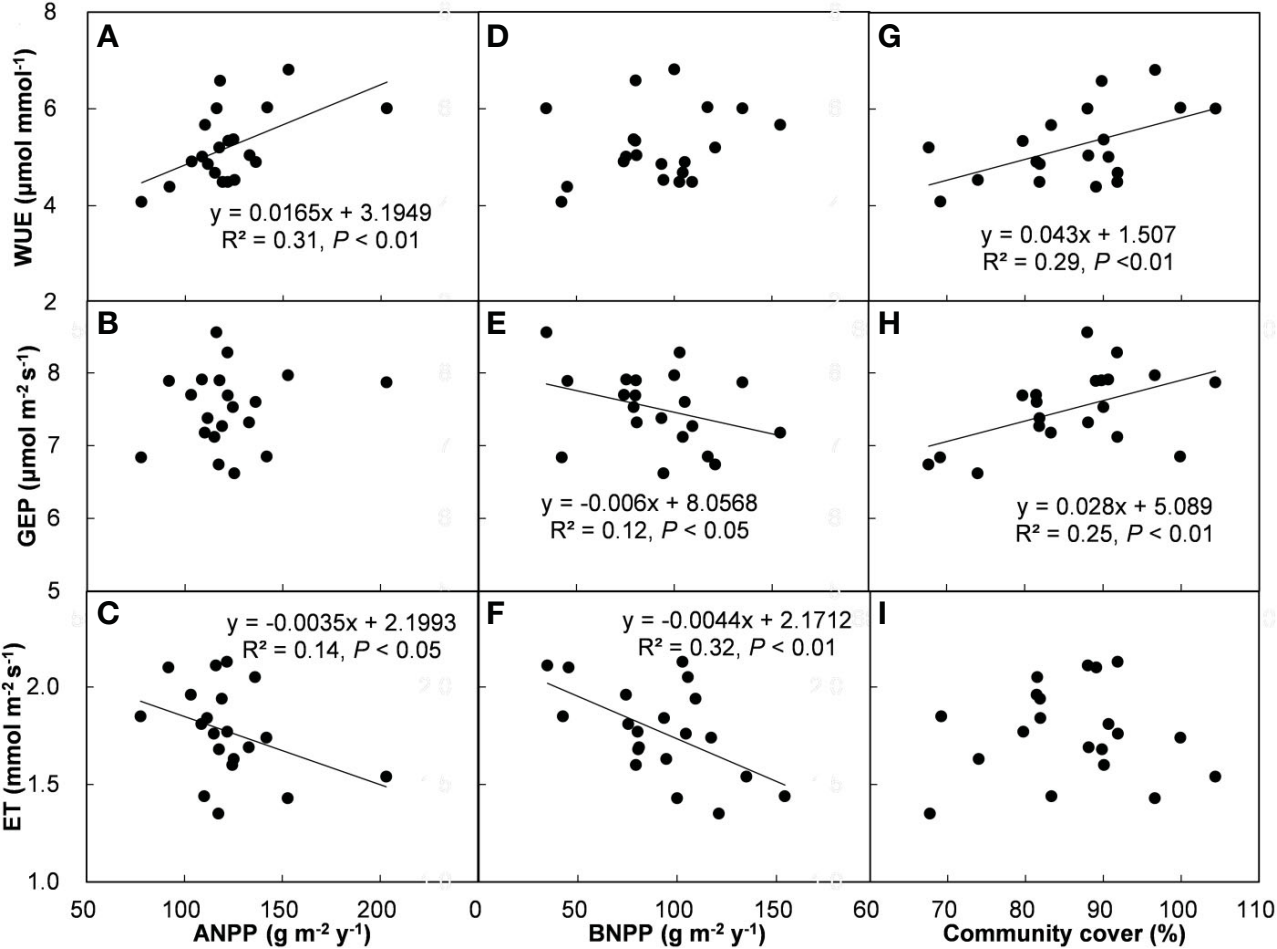
Relationships of WUE, GEP, and ET with ANPP **(A–C)**, BNPP **(D–F)**, and community cover **(G–I)**.

Across all the treatments and years, ecosystem WUE and GEP had positive correlations with SSM (**Figure 5D, E**), but there was no relationship between ET and SSM (**Figure 5F**). In addition, ecosystem WUE, GEP, and ET were unrelated to FSM (**Figures 5A–C**) and SM (**Figures 5G–I**). Across all the plots, ecosystem WUE showed a positive linear correlation with ANPP and community cover (**Figures 6A, G**, both *P* < 0.01), but not related to BNPP (**Figure 6D**). GEP showed a negative correlation with BNPP (**Figure 6E**, *P* < 0.05), a positive correlation with community cover (**Figure 6H**, *P* < 0.01), but no correlation with ANPP (**Figure 6B**). ET was negatively related to ANPP and BNPP (**Figures 6C, F**, both *P* < 0.01), but was not significantly influenced by community cover (**Figure 6I**).

## Discussion

### Effects of FP+ on WUE

Precipitation in the first half of growing season is critical for plant growth (Chelli et al., 2016). Higher first half of growing-season precipitation has been reported to enhance plant productivity (Bates et al., 2006; Peng et al., 2013; Denton et al., 2017). A wetter early growing season can promote plant activity and leaf development, which may subsequently increase C and water fluxes between leaves and the atmosphere through stomata. However, the anticipated positive effect of FP+ on ecosystem WUE did not occur in our study, is due to tiny change in both GEP and ET in the FP+ treatment.

The lack of significant response of WUE to FP+ in this study is inconsistent with the increase in WUE in a meadow steppe (Dong et al., 2011). Differ climate conditions and soil water storage capacity between the two sites may explain the differences in responsiveness of WUE. On one hand, the lower temperature and weaker solar radiation during the first half of growing season at our study site may hinder plant growth, and the added rainwater cannot effectively promote C sequestration. On the other hand, lower water storage capacity of sandy soil in the study site (Niu et al., 2011) and the abundance of immature plant roots in the first half of growing season mean that rainwater is ineffectively intercepted and absorbed, and much of the rainwater may rapidly infiltrate into deeper soil. Most plants are shallow-rooted, additional water seeping into deeper soils may not be utilized by plants (Ru et al., 2018). In contrast, plants in the meadow steppe have more developed root systems and the clay soil has higher water storage capacity, and thus the increased spring rainfall would be absorbed and utilized by plants and stimulate C sequestration.

### Effects of SP+ on WUE

Water is a strong controlling factor of primary productivity, particularly in ecosystems with little water availability (Ru et al., 2018; Zhang et al., 2021). Across ecosystems, precipitation seasonality can forecast plant community productivity more accurately than precipitation amount (Robinson et al., 2013). In our study, a large increase in ecosystem WUE under the SP+ treatment resulted from an increase in GEP but no effect on ET. SP+ significantly enhanced SSM, while SSM was positively correlated with GEP but not with ET. At this study site, root growth reached peak in August. Therefore, the alleviation of water stress during the second half of growing season ensured plants could fully exploit water and nutrients. Meanwhile, temperature and solar radiation, which can directly stimulate leaf area and GEP through their promotion effects on photosynthetic area and capacity (Guerrieri et al., 2016) and indirectly enhance leaf stomatal conductance and nutrient supply (Zhang et al., 2017), are better in the second half than in the first half of growing season, which. In addition, the well-developed roots of the second half of growing season are more capable of absorbing water. Together, these factors produced favorable conditions for plant growth and microbial activity, which can enhance water and nutrient acquisition (Trivedi et al., 2020), and thus accelerated plant gas exchange and subsequently exacerbated the promotion effect of increasing precipitation on C input in comparison with other periods.

Nevertheless, elevated second half of growing-season water supply did not stimulate ET. One possible reason is that ET includes the water fluxes from both plant canopy transpiration and soil evaporation (Nie et al., 2021). Higher precipitation during second half of growing season could enhance plant community cover and stomatal conductance, which leads to greater canopy transpiration and photosynthesis. Meanwhile, great canopy cover would reduce exposure of bare soil, and subsequently suppress soil evaporation (Zheng et al., 2019). At the ecosystem scale, the increase in canopy transpiration may offset the decrease in soil evaporation, resulting in no net change in ET under SP+ treatments.

The greater dependence of GEP rather than ET on second half of growing season water supply supports previous results showing that the impact of precipitation on ecosystem WUE is determined by C processes rather than water processes (Reichstein et al., 2002; Niu et al., 2011; Zhang et al., 2017; Bai et al., 2020). This pattern is consistent with observations that ecosystem WUE enhanced with increasing precipitation in semiarid zones (Niu et al., 2011; Zhang et al., 2018; Bai et al., 2020). The positive dependence of ecosystem WUE on ANPP and plant community cover provide further support for the above argument (**Figure 4**). Increases in plant community cover can enhance photosynthetic area. Along with increased photosynthesis, plants can proportionally uptake more C, and finally led to higher GEP under SP+ treatments. This semiarid grassland is dominated by herbaceous plants, whose metabolic activities are strongly dependent on soil water availability. During the 4-yr study period, the second half of growing season accounted for 73.5% of entire growing-season precipitation, so increasing the precipitation magnitude of second half of growing season may alleviate water stress more effectively than that of first half of growing season. A field experiment that shifted the timing of growing-season precipitation peak in this grassland demonstrated that precipitation amount in July and August was more important in regulating C release than any other period in the growing season (Ru et al., 2018). In this study, July, August, and September were the second half of growing season. The synchronization of greater soil water availability, higher temperature and stronger solar in this period could stimulate the growth of herbaceous plants and the metabolism of microbial enzyme, and subsequently contribute to increase in ecosystem WUE.

Second half of growing-season precipitation contributed to the majority of entire growing-season precipitation, the promotion effect of SP+ on soil water availability may last for a long time, it may even extend into the next year. However, the sandy soil of the study site cannot store too much water, most unutilized water would be lost, SP+ only slightly increased FSM. Meanwhile, FSM had no correlation with GEP, ET, or ecosystem WUE. Therefore, the legacy effect of SP+ on first half of growing-season C and water cycles can be ignored.

### Implications for ecosystem WUE under shifting precipitation

Our findings provide valuable implication for forecasting ecosystem C and water fluxes in response to shifting precipitation in semiarid grasslands. With increasing frequency of extreme precipitation events, the changes in C sequestration and water loss are predicted to cause corresponding change in ecosystem WUE because C sequestration and water loss respond differently to precipitation timing in the growing season.

Increasing precipitation in the second half of growing season presented a larger positive influence on ecosystem WUE than increasing precipitation in the first half of growing season. Enhancing water availability in the second half of growing season increased GEP but did not affect ET, resulting in an increase in ecosystem WUE. In our study site, the second half of growing-season precipitation was much more than the first half of growing-season precipitation in our study site, which may exaggerate the effects of changing precipitation at fixed ratio, so if we want to verify the effects of precipitation timing on WUE, we need to design more reasonable experiments to consider both precipitation amount and timing. For example, we can choose areas where or years when the first half and second half of growing-season precipitation amounts are close.

## Conclusions

Ecosystem WUE is an important metric linking plant physiological processes and environmental change. A better forecasting of how ecosystem WUE respond to shifting precipitation regimes and their intrinsic driving mechanism will help clarify how ecosystems adapt to ongoing climate change. Using a 4-yr field experiment in a semiarid temperate steppe in Northern China, we examined the impact of increasing precipitation magnitude at different periods of growing season on ecosystem WUE and its components. Although increasing first half of growing season precipitation had little effect on ecosystem WUE and its components, increasing second half of growing season precipitation enhanced ecosystem WUE by stimulating C assimilation process (GEP) but with no changes in water loss process (ET). The relationship between ecosystem WUE and precipitation amount at different periods in the growing season indicated that the temperate steppe in Mongolian Plateau may sequester C more effectively when there is ample water in the second half of growing season. These findings provide key insights into the consequences of shifting precipitation regimes and acquire a more thorough cognition of C and water cycles.

## Data availability statement

The original contributions presented in the study are included in the article/supplementary material. Further inquiries can be directed to the corresponding author.

## Author contributions

JZ: Data curation, writing-original draft preparation. ZY: Methodology, supervision. DQ: Visualization, investigation. LS: Conceptualization, writing-reviewing and editing.
